# Impact of Ventilation on Respiratory Virus Transmission in College Residence Hall Cohorts: Potential for Causal Inference About Mode of Transmission

**DOI:** 10.1111/irv.70133

**Published:** 2025-07-02

**Authors:** T. Louis Gold, Kathleen M. McPhaul, Huang Lin, Ryan Doughty, Irina Maljkovic Berry, Filbert Hong, Jianyu Lai, Todd J. Treangen, Jelena Srebric, Donald K. Milton

**Affiliations:** ^1^ Department of Global, Environmental, and Occupational Health University of Maryland School of Public Health College Park Maryland USA; ^2^ Department of Epidemiology and Biostatistics University of Maryland School of Public Health College Park Maryland USA; ^3^ Department of Computer Science Rice University Houston Texas USA; ^4^ Viral Diseases Branch Walter Reed Army Institute of Research Silver Spring Maryland USA; ^5^ Department of Bioengineering Rice University Houston Texas USA; ^6^ Department of Mechanical Engineering University of Maryland School of Engineering College Park Maryland USA

**Keywords:** causal analysis, cohort study, coronaviruses, dormitory, epidemiology, influenza, respiratory virus transmission, ventilation

## Abstract

**Background:**

The SARS‐CoV‐2 pandemic focused attention on airborne‐inhalation transmission and building ventilation. However, investment in solutions lags because few epidemiologic studies demonstrate a causal effect of ventilation on acute respiratory infection (ARI) transmission. This highlights a need for improved study designs to support causal inference.

**Methods:**

To investigate the potential for causal inference, we analyzed prospective cohorts residing in a high‐ventilation (HVent, ≥ 5 L/s per person) or a neighboring low‐ventilation (LVent, < 5 L/s per person) college residence hall during two spring semesters (2018 and 2019). Swab samples, analyzed using a PCR panel for respiratory pathogens, were collected based on self‐reported symptoms and contacts. Our analysis focused on roommate pairs where both had been tested within a 2‐week period. Roommate pairs with concordant positive PCR results were categorized as possible transmission events. We used genetic sequencing and phylogenetic analysis to identify probable transmission clusters and events.

**Results:**

We analyzed data from 368 cohort participants (82 HVent and 286 LVent), including 60 person‐infections, with a trend toward 54% lower ARI risk among students living in HVent versus LVent residence halls. We identified 97 roommate pairs, 64 two‐week intervals where both members were tested, 36 (2 HVent and 34 LVent) intervals with ≥ 1 infection, and four possible transmission events (all LVent). Sequence data available for two of the four events confirmed one probable transmission cluster and one probable transmission event.

**Conclusions:**

Future college dorm transmission studies should prioritize enrolling roommates rather than individuals, measuring ventilation, and confirming transmission events through whole genome sequencing.

## 1 Introduction

In the wake of the COVID‐19 pandemic, ASHRAE, the United States Centers for Disease Control and Prevention, and the World Health Organization released documents supporting the use of increased ventilation to control the transmission of acute respiratory infections (ARIs) [1, 2, 3]. However, political will and financial resources to implement the guidelines are far from assured in the absence of prospective human studies clearly demonstrating causality and utility outside of pandemic emergencies.

The current evidence base for inhalation transmission of seasonal influenza and other non‐pandemic viruses relies heavily on modeling studies of indoor ARI transmission [4, 5, 6, 7]. Other sources include laboratory exposure mitigation studies and epidemiologic studies of ventilation and ARI that focus on infection risk rather than transmission and are largely ecological [8, 9, 10]. Here, we use data from a natural experiment and prospective college cohort living in high‐ventilation (HVent) and low‐ventilation (LVent) buildings to inform an improved prospective cohort study design for supporting causal inference about the contribution of airborne‐inhalation transmission and the impact of transmission in future investigations.

## 2 Methods

The data from the cohort study used for this analysis were published in part by Adenaiye et al. [11] and Zhu et al. [12]. Here, we present 2 years of data and a new approach to the analysis with implications for a new prospective observational study design. The University of Maryland Institutional Review Board and the Human Research Protection Office of the Department of the Navy approved this study. All participants provided informed consent.

Dynamic cohorts of college students residing in an HVent (≥ 5 L/s per person) or a neighboring LVent (< 5 L/s per person) residence hall were enrolled each year for three consecutive academic spring semesters (2018–2020) and monitored prospectively for ARI. Building environmental conditions and ventilation characteristics were monitored and characterized as described by Zhu et al. [12]. The data presented here are from the spring semesters of the 2018 (Year 1) and the 2019 (Year 2) academic years. Participants provided residence hall name, room number, sex, age, and hours per day spent in their room in a baseline questionnaire.

The cohort study design, including recruitment and testing of cases and their contacts, was previously described [11]. Briefly, cases were identified via daily symptom questionnaires, and both the symptomatic case and each person in the cohort whom the case reported having spent time with in the previous 24 h (contacts) were tested for a large panel of respiratory pathogens (TaqMan Array Card, Thermo Fisher Scientific). This analysis focuses on case and contact pairs that were roommates.

Participants rated their symptoms on a scale from 0 to 3 (0 = *no symptoms*, 1 = *just noticeable*, 2 = *clearly bothersome from time to time but didn't stop me from participating in activities*, 3 = *quite bothersome most or all of the time and stopped me from participating in activities*), which were then aggregated into four categories, as previously described: upper respiratory, lower respiratory, systemic, and gastrointestinal [13].

If a participant tested positive multiple times, we assigned Day 0 of infection for each pathogen as the day of first sample collection with a positive PCR test. Any individual's subsequent positive tests for a pathogen occurring more than 30 days after the last available positive test for that pathogen were classified as new infections and assigned a new Day 0. We counted dual infections as two separate person‐infections. We calculated person‐infections (each unique viral infection per participant) and cumulative infection risk (person‐infections as a percentage of cohort participants) for both buildings.

We identified enrolled roommate pairs (persons living in triples or quads could contribute to more than one pair) and a transmission cohort of pairs where both participants were tested within at least one 14‐day interval. Each pair could contribute more than one pair‐surveillance interval. Transmission‐risk intervals were defined as the intervals where at least one member of the pair tested positive. Roommate pairs with concordant positive PCR results were categorized as a *possible* transmission event.

We used genetic sequencing and phylogenetic analysis similar to those described by Berry et al. [14] to identify *probable* transmission clusters and events (Supporting Information). Sequencing was performed at the Walter Reed Army Institute for Research and the Mount Sinai Icahn School of Medicine. In this report, we include all samples from the cohort containing viruses implicated in possible transmission events where there was sufficient sample and sequencing passed quality control.

We used logistic regression (glm, R Version 4.3.1) of person‐infection risk to estimate the relative risk of infection for residents of the LVent building compared to those in the HVent building, adjusting for the unbalanced gender distribution. Age was not included as a covariate, as the distribution was balanced and the range was narrow within the dormitory population (Table 1). We computed possible transmission risk as a percentage of transmission‐risk intervals. Finally, we used the infection risk and the possible transmission risk in the LVent building from Year 2 to estimate the minimum sample sizes needed to detect a range of reductions due to ventilation in future studies with 80% power and a Type I error of 0.05 (R package “pwrss,” Version 0.3.1).

**TABLE 1:** Cohort participant characteristics in high‐ and low‐ventilation buildings by year.

Residence hall ventilation	Overall	Year 1		Year 2	
		HVent	LVent	HVent	LVent
Residents	1466	200	534	198	534
Cohort participants (%)^a^	368 (25.1)	27 (13.5)	151 (28.3)	55 (27.8)	135 (25.3)
Cohort room type (%)					
Single	28 (7.6)	2 (7.4)	7 (4.6)	9 (16.4)	10 (7.4)
Double	292 (79.3)	22 (81.5)	127 (84.1)	40 (72.7)	103 (76.3)
Triple	31 (8.4)	3 (11.1)	11 (7.3)	3 (5.5)	14 (10.4)
Quad	17 (4.6)	0 (0.0)	6 (4.0)	3 (5.5)	8 (5.9)
Male (%)	173 (47.0)	15 (55.6)	63 (41.7)	39 (70.9)	56 (41.5)
Age (mean ± SD)	19.0 ± 0.59	19.2 ± 0.54	19.0 ± 0.53	19.0 ± 0.77	19.0 ± 0.55
Hours/day in room (mean ± SD)	12.1 ± 3.09	12.2 ± 2.22	11.9 ± 3.16	12.5 ± 3.11	12.5 ± 3.05

*Note:* Percentages and averages are calculated among cohort participants except as noted.

^a^ Cohort participant: Any resident who enrolled and completed a baseline questionnaire (% of residents).

## 3 Results

During the study period, 1466 students lived in two neighboring residence halls, a smaller HVent (398) and a larger LVent (1068) building, of whom 368 enrolled as cohort participants (Table 1). Students reported spending slightly more than 12 h/day in their rooms. We detected 60 person‐infections among participants, and ARI risk trended 54% lower among students residing in the HVent building than in the LVent building over the course of two successive resident cohorts (Table 2). Infection risk averaged 8.5% in HVent and 18.5% in LVent. Based on the logistic regression model of person‐infection risk, the odds of infections attributable to LVent are 1.4 (95% confidence interval 0.65–3.3). Coronaviruses were the most frequently detected ARIs, followed by influenza viruses and RSV (Table 2).

**TABLE 2:** Participant role, symptoms, and ARI incidence in high‐ and low‐ventilation buildings by year.

Residence hall ventilation	Overall (*N* = 368)^a^	Year 1		Year 2	
		HVent (*N* = 27)	LVent (*N* = 151)	HVent (*N* = 55)	LVent (*N* = 135)
Case participants)^b^					
Individuals	52	1	25	5	21
Enrollments	54	1	27	5	21
Contact participants)^c^					
Individuals	87	1	50	5	31
Enrollments	101	1	56	7	37
Never case or contact	254	25	90	47	92
Symptom scores)^d^					
Median upper respiratory (IQR)	5.0 (3.0–6.0)	7.0 (0.0–0.0)	5.0 (3.5–6.5)	4.0 (2.0–6.0)	4.0 (2.0–6.0)
Median lower respiratory (IQR)	2.0 (1.0–3.0)	4.0 (0.0–0.0)	2.0 (1.0–3.0)	2.0 (1.0–3.0)	2.0 (1.0–3.0)
Median systemic (IQR)	2.0 (1.0–4.8)	3.0 (0.0–0.0)	3.0 (1.5–6.5)	3.0 (1.0–3.0)	1.0 (0.0–4.0)
Median gastrointestinal (IQR)	0.0 (0.0–0.8)	0.0 (0.0–0.0)	0.0 (0.0–1.0)	0.0 (0.0–0.0)	0.0 (0.0–0.0)
Temperature (°C), mean ± SD	37.2 ± 0.6	37.1 ± 0.0	37.3 ± 0.7	36.9 ± 0.2	37.2 ± 0.4
Person‐infections)^e^	60	1	31	6	22
Infection risk)^f^	16.3%	3.7%	20.5%	10.9%	16.3%
Viruses detected^g^					
CoVs	36	1	15	4	16
IAV or IBV	17	0	12	1	4
RSVA or RSVB	7	0	4	1	2

^a^ Cohort participants (see Table 1); participants may enroll multiple times as cases and/or contacts.

^b^ Case participants: PCR positive (Ct ≤ 35) cohort participant or a contact participant who becomes PCR positive within 14 days of being named by a PCR‐positive case participant.

^c^ Contact participants: Cohort participant identified by a case participant as having interacted with them in the last 24 h.

^d^ Symptom scores: Symptoms reported by case participants at the first PCR‐positive visit. Upper respiratory (max score: 15) = runny nose + stuff nose + sneeze + sore throat + earache; lower respiratory (max score: 9) = chest tightness + shortness of breath + cough; systemic (max score: 12) = malaise + headache + muscle/joint ache + sweats/fever/chills; gastrointestinal (max score: 9) = nausea + vomit + diarrhea.

^e^ Person‐infections: Each case participant contributed one observation per distinct pathogen detected.

^f^ Cumulative infection risk: Person‐infections as a percentage of cohort participants.

^g^ Viruses detected: CoVs: CoV229E, COVHKU1, CoVNL63 CoVOC43; IAV or IBV: Influenza virus A or influenza virus B; RSVA or RSVB: Respiratory syncytial virus A or respiratory syncytial virus B. Counts for each specific virus are in Table S1.

We identified 97 roommate pairs among the cohort participants (Table 3). Of these pairs, 39 were tested at least once within 14 days of each other for a total of 64 pair‐surveillance intervals. There were 36 transmission‐risk intervals where at least one pair member had a positive PCR test; coinfections were counted as two intervals. We detected four possible transmission events in 34 transmission‐risk intervals observed in the LVent building (possible transmission risk = 11.8%) and no events during the two intervals observed in the HVent building.

**TABLE 3:** ARI within roommate pairs in high‐ and low‐ventilation buildings by year.

Residence hall ventilation	Overall	Year 1		Year 2	
		HVent	LVent	HVent	LVent
Enrolled roommate pairs^a^	97	2	43	12	40
Transmission cohort^b^	39	0	18	2	19
Pair‐surveillance intervals^c^	64	0	26	3	35
Transmission‐risk intervals^d^	36	0	18	2	16
Possible transmission^e^	4	0	1	0	3
Probable transmission cluster^f^	2	0	1	0	1
Probable transmission^g^	1	0	0	0	1
Possible transmission risk^h^	11.1%	0.0%	5.6%	0.0%	18.8%
Possible transmission count^i^					
CoVs	3	0	0	0	3
IBV	1	0	1	0	0
RSVA or RSVB	0	0	0	0	0

^a^ Enrolled roommate pairs: Number of cohort participant roommate pairs. Double, triple, and quad rooms contribute, at most, one, three, and six pairs, respectively.

^b^ Transmission cohort: Unique roommate pairs where both members were tested within at least one 14‐day interval.

^c^ Pair‐surveillance intervals: Unique roommate pairs contributed one or more surveillance intervals.

^d^ Transmission‐risk intervals: Each observation represents a pair‐surveillance interval where at least one member of the pair was positive for a respiratory virus. Pair‐surveillance intervals contributed one observation per pathogen detected.

^e^ Possible transmission: Transmission‐risk intervals where the PCR results for both members of the pair matched.

^f^ Probable transmission cluster: Sequencing data and phylogenetic analyses indicate the pair is likely part of a closely related cluster. Sequencing was successful for only one of the three CoV pairs.

^g^ Probable transmission: Sequencing data and phylogenetic analyses indicate the pair is likely a direct transmission event.

^h^ Possible transmission risk: Possible transmission risk per transmission‐risk interval.

^i^ Possible transmission count: CoVs: CoV229E, COVHKU1, CoVNL63 CoVOC43; IBV: influenza virus B; RSVA or RSVB: respiratory syncytial virus A or respiratory syncytial virus B.

**FIGURE 1:**
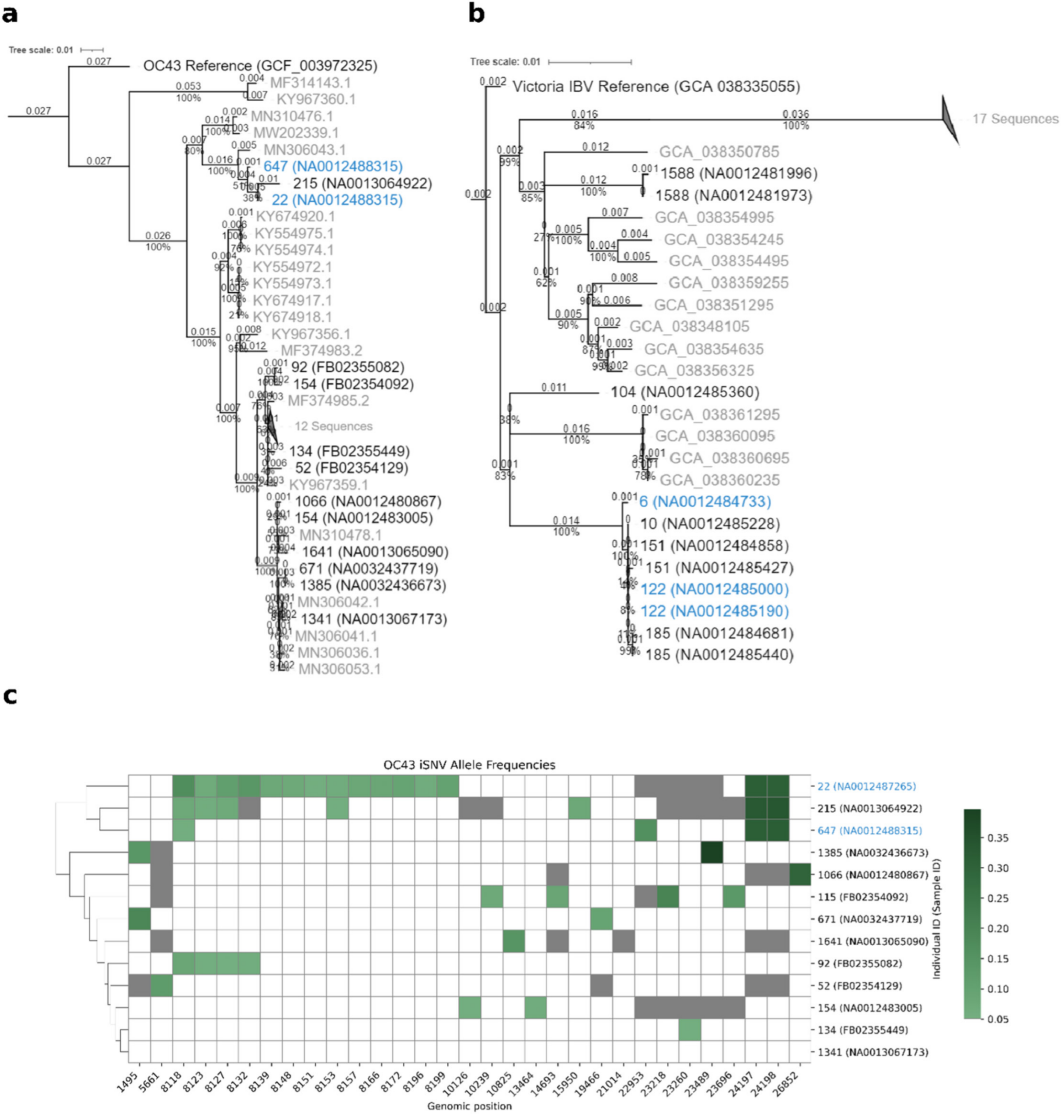
(a, b) Phylogenetic analyses of OC43 and Victoria lineages, respectively. Cohort samples are in black and are labeled with the participant ID followed by the sample ID; blue denotes roommate pairs. Gray samples are non‐cohort samples and are labeled by NCBI Nucleotide and GenBank IDs, respectively. Trees are rooted at the reference, which is labeled by the GenBank ID. Branch distances and bootstrap values are mapped onto the respective branches. (c) Intra‐host single‐nucleotide variation (iSNV) for the OC43 lineages. iSNVs were called at an allele frequency greater than 0.05 at loci with a depth greater than 100. Regions not meeting the depth threshold are indicated in gray. iSNV frequency is indicated by the color bar.

Genetic sequencing data were available for (influenza B) IBV and OC43, including two of the observed roommate pairs with possible transmission events. Sequencing of NL63 viruses, including the other possible transmission events, was unsuccessful. Of the IBV Victoria viruses, all but two samples (1588 and 104) from the cohort clustered together. However, one of the roommates (6) was outside of the main cluster, suggesting the second roommate (122) had an IBV strain closer to other individuals. We categorized this roommate pair as part of a probable transmission cluster but not a probable transmission event (Figure 1b and Table 3) [14]. Sequencing of OC43 viruses from the cohort revealed three samples, including a roommate pair, with 90 unique single‐nucleotide differences from the remaining samples. Five additional single‐nucleotide differences were shared only between the two roommates. The group of three also shared unique iSNVs in the two nucleotide loci from 24,197 to 24,198, indicating close linkage in a transmission chain. We categorized this as a probable transmission event for the roommate pair (Figure 1a,c).

**FIGURE 2:**
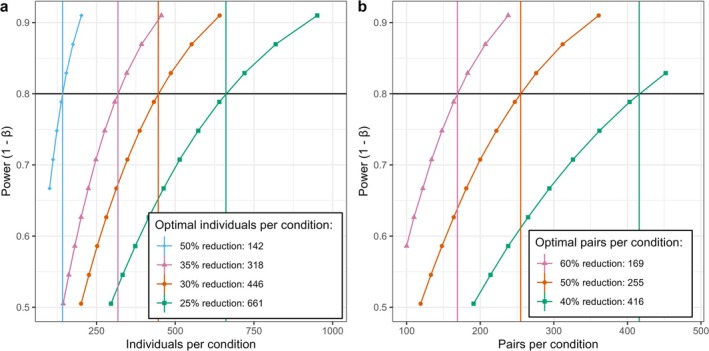
(a) Estimated number of individuals needed per ventilation condition to detect a 50%, 35%, 30%, or 25% reduction in possible risk of infection (80% power and 0.05 Type I error). (b) Estimated optimal pairs needed to achieve 80% power to detect a 60%, 50%, or 40% reduction in possible transmission risk with a Type I error of 0.05.

We estimate a future study would need a minimum of 142 individuals living in each building to detect a 50% reduction and 446 individuals in each to identify a 30% reduction of individual infection risk between HVent and LVent systems with 80% power and a Type I error of 0.05 (Figure 2a). By contrast, we estimate that 255 roommate pairs per ventilation condition would be needed to achieve 80% power to detect a 50% reduction in possible transmission risk with a Type I error of 0.05 (Figure 2b).

## 4 Discussion

In this analysis of ARI in college residence halls, we show how a prospective cohort design focusing on transmission between roommates, rather than more generally among reported close contacts, could facilitate inference about causal relationships between ventilation and respiratory virus transmission.

The original study had significant strengths including prospective collection of daily symptom reports, extensive environmental monitoring, and whole‐genome sequencing. However, it also had several limitations contributing to finding a nonsignificant result despite a trend toward a large effect size. First, recruitment drew from two buildings with substantially different population sizes (LVent: ~545 residents; HVent: ~223 residents). This resulted in an unbalanced sample and reduced statistical power. Additionally, contact enrollment relied on retrospective identification and was subject to incomplete ascertainment, potentially leading to selection bias. A larger sample, routine testing, and a more balanced design would enhance the precision and robustness of effect estimates. Second, a significant portion of overall exposure to infectious persons was likely to have occurred outside of the monitored environments and introduced the possibility of confounding and attenuation of observed associations with residence hall ventilation toward the null.

In future studies aiming to identify a causal effect of ventilation—and thereby elucidate the role of airborne or inhalation‐based transmission—a more robust design would involve the prospective identification of contact pairs whose exposure to one another occurs predominantly under potentially well‐characterized environmental conditions. We recommend prospectively enrolling roommate pairs and monitoring their room's ventilation because students reported spending half their time in their room. Although pairs may share other exposures (e.g., classes, dining halls, other dorm rooms), time spent in these other areas is minimal in comparison. Thus, bias to the null due to sharing rebreathed air outside of their room is minimized [15] and is similar for HVent and LVent roommates. Additionally, time spent with and infection by a third infected person outside of their room would be similar for students regardless of room ventilation rates. If transmission from other than their roommate occurs in their room or similar rooms in their building, it would reflect the same building ventilation‐related risk and thus not lead to bias. To address residual imbalance in baseline covariates and potential interference between units, we propose applying the inverse probability weighting (IPW) estimator developed by Tchetgen and VanderWeele [16], which accommodates causal inference in the presence of partial interference.

Based on the analysis presented here, we estimate that 255 pairs per ventilation system would give 80% power to detect a 50% reduction in possible transmission risk (Type I error of 0.05). This equates to a total of 510 individuals per system, with 80% power to detect a 28% reduction of infection risk. This power analysis is conservative. Using twice‐weekly testing of prospectively identified pairs [17], a new study would be able to detect transmission from asymptomatic cases so that a larger number of transmission‐risk intervals would be observed than estimated based on these data, thereby increasing power. Furthermore, by accounting for the baseline confounding between groups and using individual room environmental measurements as continuous exposure variables, we can increase the precision of estimates and thus reduce the sample size without sacrificing power. We acknowledge that the COVID‐19 pandemic may have produced some lasting environmental and behavioral changes not reflected in these pre‐pandemic data, such as improved ventilation in academic buildings and occasional mask wearing, that may influence incidence and transmission of respiratory viruses on college campuses. Future studies will need to take these into account.

In conclusion, we outline a college dormitory cohort study design capable of supporting causal inference about the role of airborne‐inhalation transmission and the impact of improved ventilation on ARI transmission by focusing on roommate pairs.

## Author Contributions

**T. Louis Gold:** writing – original draft, formal analysis, methodology, visualization. **Kathleen M. McPhaul:** writing – review and editing, supervision. **Huang Lin:** writing – review and editing, formal analysis, methodology, validation. **Ryan Doughty:** writing – review and editing, formal analysis, visualization. **Irina Maljkovic Berry:** investigation, writing – review and editing, formal analysis, methodology. **Filbert Hong:** data curation, project administration, writing – review and editing, investigation. **Jianyu Lai:** investigation, writing – review and editing, formal analysis, validation. **Todd J. Treangen:** investigation, writing – review and editing, formal analysis, supervision, methodology. **Jelena Srebric:** investigation, writing – review and editing, resources. **Donald K. Milton:** conceptualization, investigation, funding acquisition, writing – review and editing, project administration, supervision, resources, formal analysis.

## Disclosure

Material has been reviewed by the Walter Reed Army Institute of Research. There is no objection to its presentation and/or publication. The opinions or assertions contained herein are the private views of the authors and are not to be construed as official or as reflecting true views of the Department of the Army or the Department of Defense. The investigators have adhered to the policies for protection of human subjects as prescribed in AR 70‐25.

## Ethics Statement

This study was approved by the Institutional Review Boards (IRBs) of the University of Maryland and the Department of Navy Human Research Protections Office.

## Consent

Informed consent was obtained from each participant. All documentation was collected electronically.

## Conflicts of Interest

D.K.M. reports consulting for AIR LLC.

## Peer Review

The peer review history for this article is available at https://www.webofscience.com/api/gateway/wos/peer‐review/10.1111/irv.70133.

## Supporting information


**Table S1** Total counts of detected viruses in high and low ventilated buildings by year.
**Table S2** Roommate pair genome sequence status by sequencing lab.
**Table S3** Chronology of detection for sequenced pathogens.

## Data Availability

Deidentified data can be accessed in the public repo at https://osf.io/jwvsz/.
