# Supplementary material for: Impact of Ventilation on Respiratory Virus Transmission in College Residence Hall Cohorts: Potential for Causal Inference About Mode of Transmission
Source: Influenza Other Respir Viruses. 2025 Jul 2;19(7):e70133. doi: 10.1111/irv.70133 (PMC12221802; doi:10.1111/irv.70133)
Supplement: Supplementary file 1 — Table S1 Total counts of detected viruses in high and low ventilated buildings by year. Table S2 Roommate pair genome sequence status by sequencing lab. Table S3 Chronology of detection for sequenced pathogens. [file IRV-19-e70133-s001.docx]

# Supplementary Information

## Supplementary Methods

For this analysis, we defined case participants as cohort participants who tested positive on a PCR sample (Ct≤35) for coronaviruses 229E, HKU1, NL63, OC43, influenza viruses A or B (IAV, IBV), or respiratory syncytial virus A or B.

Additional contemporary sequences for both Victoria and OC43 lineages were downloaded from the NCBI Virus Genome Explorer. Victoria samples were selected by querying for complete assemblies of *Betainfluenzavirus influenza* (taxid:2955465) from DC and Maryland with a collection date from Jan 1, 2018 – Dec 31, 2018. The query returned 82 assemblies, including those from this study. After downstream phylogenetic analyses, 41 samples were removed due to dissimilarity to the cohort samples. OC43 samples were selected by querying for complete nucleotide entries of *Human coronavirus OC43 (HcoV-OC43)* (taxid:31631) in North America with collection dates from Jan 1, 2017 – Dec 31, 2019. The query returned 32 additional OC43 sequences, all of which were included in the final phylogenetic analyses.

All sequences in each lineage were aligned using Parsnp 2.0.^1^ The phylogenetic analysis was performed using ModelTest-NG^2^ to select the best-fit model, followed by tree reconstruction with RAxML^3^ using the best fit model for each lineage. Bootstrap analysis with 100 replicates was conducted to assess clade support, and bootstrap values were mapped onto the tree. Visualization was done using iTOL.^4^ An analysis of the intra-host single nucleotide variation (iSNV) analyses was performed on the variant calling output from vRAPID (<https://github.com/BakelLab/vRAPID>)

We defined ‘unique’ mutations for both single nucleotide differences and iSNVs as those contained within a set of cohort participants, and none of the rest. This follows the assumption that samples that share unique mutations are more probable to be closely related in a transmission network.

## Supplementary References

1. Bryce Kille, Michael G Nute, Victor Huang, Eddie Kim, Adam M Phillippy, Todd J Treangen, Parsnp 2.0: scalable core-genome alignment for massive microbial datasets, Bioinformatics, Volume 40, Issue 5, May 2024, btae311, https://doi.org/10.1093/bioinformatics/btae311

2. Diego Darriba, David Posada, Alexey M Kozlov, Alexandros Stamatakis, Benoit Morel, Tomas Flouri, ModelTest-NG: A New and Scalable Tool for the Selection of DNA and Protein Evolutionary Models, Molecular Biology and Evolution, Volume 37, Issue 1, January 2020, Pages 291–294, https://doi.org/10.1093/molbev/msz189

3.Alexandros Stamatakis, RAxML version 8: a tool for phylogenetic analysis and post-analysis of large phylogenies, Bioinformatics, Volume 30, Issue 9, May 2014, Pages 1312–1313, https://doi.org/10.1093/bioinformatics/btu033

4. Ivica Letunic, Peer Bork, Interactive Tree of Life (iTOL) v6: recent updates to the phylogenetic tree display and annotation tool, Nucleic Acids Research, Volume 52, Issue W1, 5 July 2024, Pages W78–W82, https://doi.org/10.1093/nar/gkae268

## Prometheus-UMD investigators:

Addo, Kofi

Adenaiye, Oluwasanmi Oladapo

Agrawala, Agrawala

Aiello, Allison

Albert, Barbara

Arria, Amelia

Bueno de Mesquita, P. Jacob

Cai, Mara

Chen, Shuo

Chen, Wilbur

Corrada Bravo, Hector

Elworth, Leo

Felgner, Philip

Frieman, Matthew

German, Jennifer

Heidarinejad, Mohammad

Hong, Filbert

Jiang, Chengsheng

Khan, Saahir

Lai, Jianyu

Liu, Hongjie

Ma, Tianzhou

Maljkovic Berry, Irina

Martinello, Richard

Mattise, Nick

Memon, Atif

Milton, Donald

Mongodin, Emmanuel

Nasko, Dan

Pop, Mihai

Porter, Adam

Romo, Sebastian

Srebric, Jelena

Tai, Sheldon

Treangen, Todd

Wajid, Faizan

Washington-Lewis, Rhonda

Wu, Qiong

Xing, Yishi

Youssefi, Somayeh

Zhu, Shengwei

## Table S1

***Total counts of detected viruses in high and low ventilated buildings by year***

| **Residence Hall Ventilation** |  | **Year 1** | | **Year 2** | |
| --- | --- | --- | --- | --- | --- |
|  | **Overall** | **Hvent** | **LVent** | **Hvent** | **LVent** |
| CoVs |  |  |  |  |  |
| CoV229E | 11 | 0 | 5 | 3 | 3 |
| CoVHKU1 | 10 | 1 | 7 | 0 | 2 |
| CoVNL63 | 6 | 0 | 0 | 1 | 5 |
| CoVOC43 | 9 | 0 | 3 | 0 | 6 |
| Flus |  |  |  |  |  |
| IAV | 9 | 0 | 4 | 1 | 4 |
| IBV | 8 | 0 | 8 | 0 | 0 |
| RSVs |  |  |  |  |  |
| RSVA | 1 | 0 | 0 | 0 | 1 |
| RSVB | 6 | 0 | 4 | 1 | 1 |
| Counts of PCR positive (Ct≤35) detections among enrolled cases and contacts residing in either high or low ventilation buildings during the study period | | | | | |

## Table S2

***Roommate pair genome sequence status by sequencing lab***

| Participant ID | Room | Sample ID | Collection Date | Sequencing Date | Organism | Sequence Location | Accession Number | GenInfo ID |
| --- | --- | --- | --- | --- | --- | --- | --- | --- |
| WRAIR | | | | | | | | |
| 6 | A | NA0012484733 | 5/3/2018 | 11/8/2018 | IBV | GenBank | MZ453306.1 | 2058304809 |
| 122 | A | NA0012485000 | 5/7/2018 | 11/8/2018 | IBV | GenBank | MZ453338.1 | 2058304885 |
| Mt. Sinai | | | | | | | | |
| 22 | B | NA0012487265 | 2/15/2019 | 05/03/2024 | CoVOC43 | GenBank | --- | --- |
| 647 | B | NA0012488315 | 2/13/2019 | 05/03/2024 | CoVOC43 | GenBank | --- | --- |
| 72 | C | NA0012488146 | 2/8/2019 | 05/03/2024 | CoVNL63 | Failed QC | --- | --- |
| 1493 | C | NA0012488250 | 2/11/2019 | 05/03/2024 | CoVNL63 | Failed QC | --- | --- |
| 1238 | D | NA0012487842 | 2/7/2019 | 05/03/2024 | CoVNL63 | Failed QC | --- | --- |
| 1259 | D | NA0012488166 | 2/12/2019 | 05/03/2024 | CoVNL63 | Failed QC | --- | --- |
| All participants resided in low ventilation building | | | | | | | | |

## Table S3

***Chronology of detection for sequenced pathogens***

| Participant ID | Building Ventilation | Sample Date | Sample ID |
| --- | --- | --- | --- |
| Y0 - CoVOC43 | | | |
| 92 | LVent | 3/3/2017 | FB02355082 |
| 52 | LVent | 3/11/2017 | FB02354129 |
| 115 | LVent | 3/13/2017 | FB02354092 |
| 134 | LVent | 3/15/2017 | FB02355082 |
| Y1 - Flu B | | | |
| 1588 | LVent | 3/30/2018 | NA0012481996 |
| 104 | --- | 5/1/2018 | NA0012485360 |
| 6 | LVent | 5/3/2018 | NA0012484733 |
| 122 | LVent | 5/7/2018 | NA0012485000 |
| 122 | LVent | 5/8/2018 | NA0012485190 |
| 10 | LVent | 5/9/2018 | NA0012485228 |
| 151 | LVent | 5/10/2018 | NA0012484858 |
| 185 | LVent | 5/13/2018 | NA0012484681 |
| Y1 - CoVOC43 | | | |
| 154 | LVent | 3/14/2018 | NA0012483005 |
| 1066 | LVent | 4/6/2018 | NA0012480867 |
| Y2 - CoVOC43 | | | |
| 647 | LVent | 2/13/2019 | NA0012488315 |
| 22 | LVent | 2/15/2019 | NA0012487265 |
| 215 | --- | 3/6/2019 | NA0013064922 |
| 1641 | LVent | 3/6/2019 | NA0013065090 |
| 1341 | --- | 4/23/2019 | NA0013067173 |
| Y3 - CoVOC43 | | | |
| 671 | LVent | 11/25/2019 | NA0032437719 |
| 1385 | --- | 12/4/2019 | NA0032436673 |
| Missing building ventilation denotes living in neither HVent nor LVent | | | |
| Blue denotes roommate pairs | | | |
